# Genetic Insights Into Early Pregnancy Loss: A Case Study of Trisomy 22 and an Enlarged Yolk Sac

**DOI:** 10.7759/cureus.67360

**Published:** 2024-08-21

**Authors:** Dipak Kolate, Nikita Bhattacharjee, Prashant Suryarao, Swapnali Sansare

**Affiliations:** 1 Obstetrics and Gynaecology, Dr. D. Y. Patil Medical College, Hospital and Research Centre, Dr. D. Y. Patil Vidyapeeth (Deemed to be University), Pune, IND

**Keywords:** qf pcr, dna microarray, early pregnancy loss, yolk sac, trisomy 22

## Abstract

The first trimester of pregnancy is crucial for organ development but also carries a high risk of complications, with early pregnancy loss being the most common. Anomalies in the yolk sac, the first extra-embryonic structure seen by ultrasonography, can indicate severe fetal growth abnormalities and are linked to higher rates of first-trimester loss. This case report details a 38-year-old woman with a history of recurrent pregnancy loss (RPL) who presented with per vaginal bleeding and mild abdominal pain. Transvaginal ultrasonography revealed a yolk sac larger than 10 mm, prompting further genetic investigation. Chromosomal microarray analysis confirmed Trisomy 22. The presence of an enlarged yolk sac, correlated with Trisomy 22, highlights the importance of early detection through sonography and genetic testing. This approach aids in managing RPL by identifying genetic causes, thereby informing pre-conception counseling and future pregnancy management. An abnormal yolk sac size necessitates thorough evaluation, including cytogenetic microarray testing and quantitative fluorescent-polymerase chain reaction analysis, to guide clinical decisions and improve pregnancy outcomes.

## Introduction

The first trimester of pregnancy is crucial for organogenesis but is also associated with a high rate of complications. Early pregnancy loss is defined as a “nonviable intrauterine pregnancy with either an empty gestational sac or a gestational sac containing an embryo or fetus without fetal heart activity before 12 weeks and 6 days of gestation.” It affects 10% of pregnancies that are spontaneously conceived and roughly 30% of pregnancies that are the result of assisted reproduction, making it the most common early pregnancy complication [1,2].

The first extraembryonic structure to be seen by ultrasonography within the gestational sac is the yolk sac. During the embryonic organogenesis stage, it is essential for providing nutritional, metabolic, endocrine, immunological, and hematopoietic support [3]. The diameter of the yolk sac typically falls between 3 and 5 mm and continues to decrease until the 10th or 11th week of pregnancy [4].

Severe growth abnormalities in the fetus are indicated by an abnormal yolk sac morphology or by a size larger than 9 mm. Furthermore, a threefold greater risk of first-trimester loss is associated with pregnancies with a mean yolk sac width of 5 mm or larger in the first-trimester ultrasound, regardless of maternal risk characteristics such as age, body mass index, polycystic ovarian syndrome, smoking, and diabetes [5].

Here, we present an interesting case of a large yolk sac associated with an abnormal karyotype.

## Case presentation

A 38-year-old, gravida 4, para 1, living 1, abortion 2, came to our private clinic with complaints of vaginal bleeding and mild abdominal pain for two days. All vital parameters were checked and found to be within normal limits (pulse: 86 beats per minute; BP: 120/80 mmHg; respiratory rate: 14 per minute; SpO2: 99% on room air). Her history included two spontaneous abortions during the first trimester. No previous medical or surgical history was noted.

**Figure 1:**
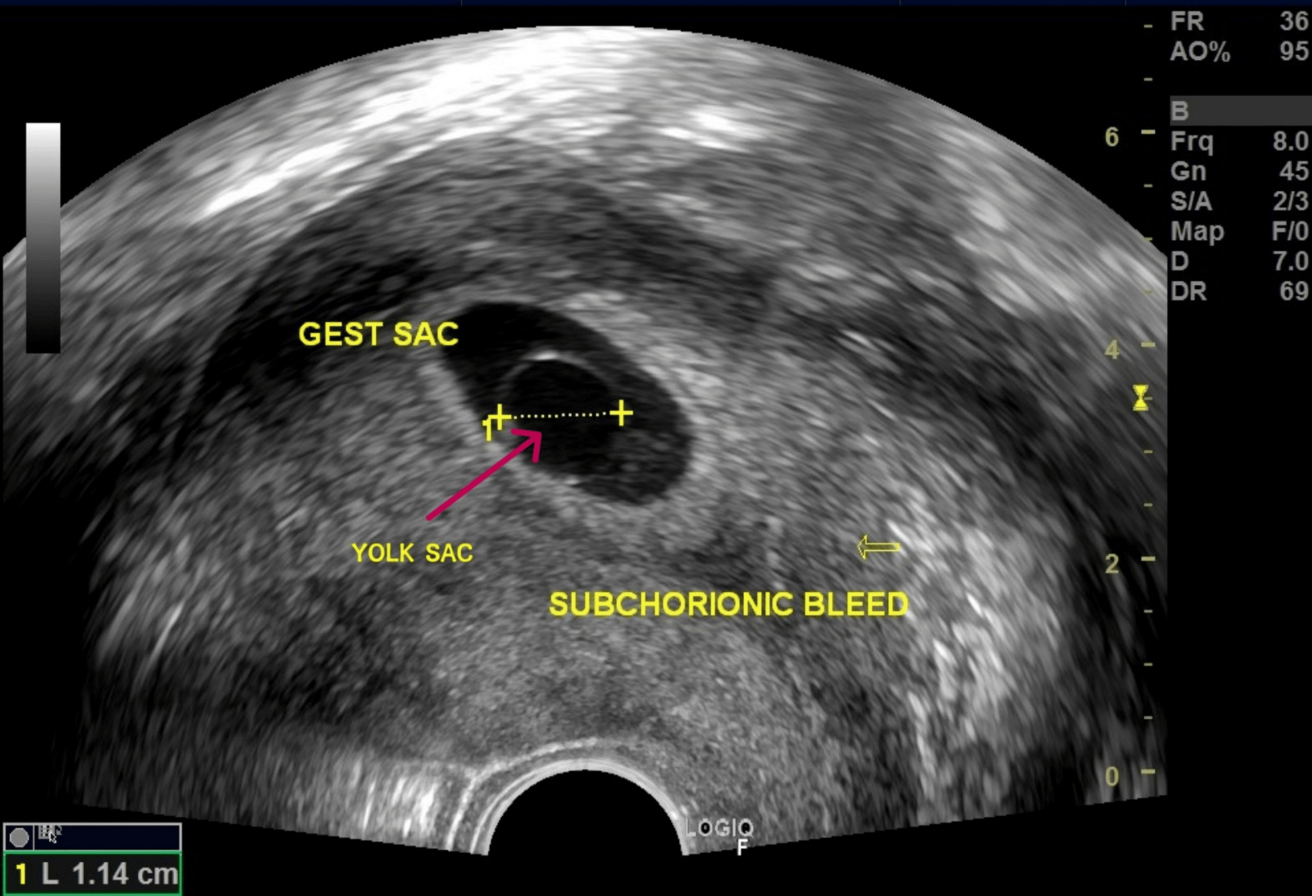
A large yolk sac measuring 1.14 cm

A transvaginal ultrasound (TVS) was performed to assess the status of the fetus. The mean sac diameter (MSD) was 2.3 cm, corresponding to 6.6 weeks of gestation, and the crown-rump length was 1.42 cm, corresponding to 7.5 weeks of gestation. The fetal heart rate was absent. The notable finding was a yolk sac measuring greater than 10 mm, which is larger than expected for the gestational age, as seen in Figure 1.

The case was diagnosed as a missed abortion, leading to the decision to proceed with surgical intervention. A dilatation and evacuation (D&E) procedure was subsequently scheduled for the patient. A decision was made to send the products of conception for chromosomal microarray analysis (CMA) and quantitative fluorescent-polymerase chain reaction (QF-PCR) analysis of common aneuploidies, such as Trisomy 13, 18, 21, and sex chromosome aneuploidies, in light of the ultrasonography findings and history of recurrent pregnancy loss (RPL) with suspected genetic abnormality.

**Figure 2:**
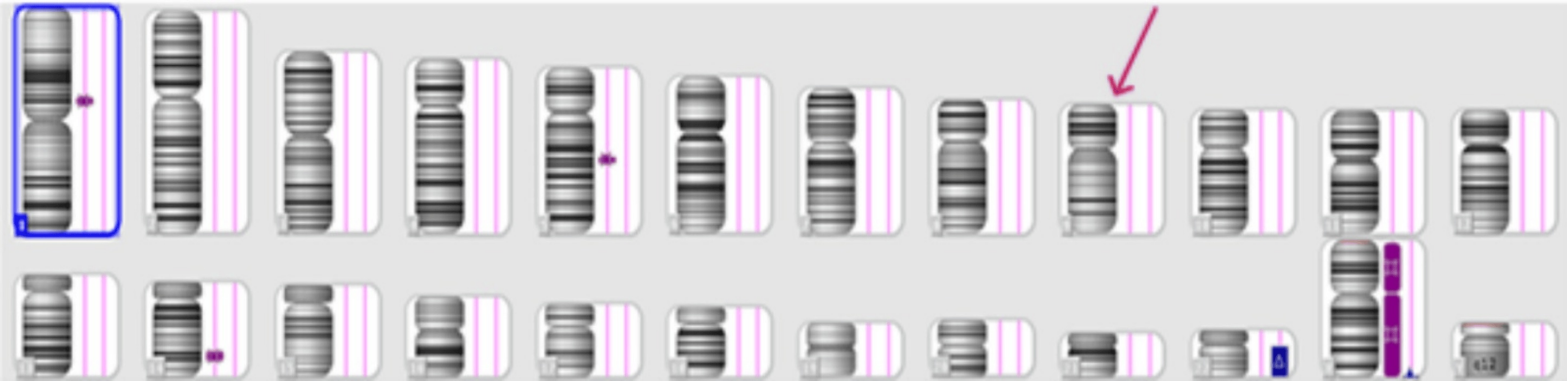
Genomic view of duplication/deletion in the analyzed DNA sample

Products of conception (POC) CMA results showed the presence of Trisomy 22 and a male sex chromosome complement. The International System for Human Cytogenetic Nomenclature (ISCN) notation was arr[GRCh37]22q11.1q13.33 (16888899_51197838)x3, seen on the cytoband q11.1-q13.33, thus pathogenic in nature (Figure 2).

CMA was performed using an Affymetrix CytoScan 315K array. This microarray consists of 315K oligonucleotide probes across the genome, including 18K unique copy number (CN) probes and 148K bi-allelic single nucleotide polymorphism (SNP) probes. Data were analyzed using Chromosome Analysis Suite (ChAS) version 4.2.1. The analysis was based on the human reference genome (GRCh37/hg19).

## Discussion

Early pregnancy losses increase the psychological and economic burden, especially for elderly gravida with a history of RPL, such as seen in our case, and are most often conceived with assisted reproductive techniques (ARTs).

Approximately 50% of early pregnancy losses are attributed to chromosomal abnormalities [6]. Among these, chromosomal trisomy is the most prevalent, accounting for 61.2% of such anomalies leading to early miscarriage. Trisomy 22 is the third most common trisomy associated with spontaneous abortions, occurring in 11-16% of cases [7].

People with Trisomy 22 seldom live, and even when they do, it is usually not in a complete, non-mosaic form. Severe organ defects are the cause of this, including kidney malformations, fetal growth retardation (FGR), congenital heart disease, cerebral deformities, and microcephaly. Thus, in individuals with Trisomy 22, postnatal survival and term or nearly term pregnancies are extremely rare. Additional phenotypic features of Trisomy 22 may include dysmorphic characteristics, such as a broad nasal bridge, epicanthic folds, webbed neck, cleft lip/palate, developmental delay, and genitourinary abnormalities [7].

Additionally, Angiolucci et al. observed that, in the great majority of cases of early pregnancy loss with Trisomy 22, an enlarged yolk sac was found. This correlation implied that Chromosome 22 might contain essential genes for embryonic development and that overexpression of these genes could result in potentially fatal anomalies in the embryo-fetal circulation. The buildup of fluid within the yolk sac may serve as a precursor to these anomalies. The abnormal rise in yolk sac size was noted before miscarriage in all cases of early pregnancy loss with an expanded yolk sac, demonstrating that the abnormal increase in yolk sac size cannot be attributed to postmortem events [8].

Ashoush et al. discovered that 36.8% of individuals with isolated congenital abnormalities had a large yolk sac, accounting for 63.6% of all cases with an extremely large yolk sac [9]. Furthermore, Srivastava et al. found that 77.78% of abortions were caused by an expanded yolk sac [10]. According to Angiolucci et al., morphological characteristics were frequently linked to chromosomal abnormalities in cases of early pregnancy loss [8].

According to Moradan and Forouzeshfar, yolk sacs were deemed abnormal if their diameters were less than 2 mm or more than 5 mm; if they were distorted or oval rather than round; if they displayed signs of degenerative changes; if they had a hyperechoic center or a hyper- or hypo-echogenic rim; or if there was not an equal number of yolk sacs relative to the embryos [5].

Detailed history taking, along with early diagnosis of an abnormal yolk sac by TVS, helps in decision-making for further advanced investigations such as QF-PCR and chromosomal microarray testing. In our case, a large yolk sac on TVS pointed toward an uncommon abnormality, so precise and timely sonography is necessary for predicting the further plan of action and, most importantly, preconception counseling for future pregnancies.

The exact etiology of pregnancy loss associated with enlarged sac diameter is unclear, and, nowadays, with the presence of microarray analysis, a definitive diagnosis can be made.

Thus, an approach that utilizes the genetic analysis of the products of conception could lessen the need for costly and time-consuming follow-up maternal studies to rule out alternative causes of RPL, while also assisting patients in coping with the psychological effects of miscarriage.

## Conclusions

In summary, the early detection of an abnormal yolk sac size through TVS can be a crucial marker for potential fetal abnormalities, particularly in cases of RPL. The presented case highlights how an enlarged yolk sac, coupled with genetic testing, can lead to the identification of significant chromosomal abnormalities, such as Trisomy 22. This underscores the importance of integrating detailed ultrasonographic evaluations with advanced genetic analyses, like CMA and QF-PCR. Such an approach not only aids in the accurate diagnosis of genetic anomalies but also enhances the management of RPL by informing pre-conception counseling and guiding future pregnancy planning. The identification of an abnormal yolk sac size should prompt thorough evaluation and timely genetic testing, which can offer valuable insights into the underlying causes of pregnancy loss and improve patient outcomes.
